# Probing the communication patterns of different chondrocyte subtypes in osteoarthritis at the single cell level using pattern recognition and manifold learning

**DOI:** 10.1038/s41598-023-41874-z

**Published:** 2023-09-02

**Authors:** Jiajian Wang, Caihong Liu, Litao Yang, Huixiong Chen, Mingqi Zheng, Yanbin Wan, Xiongxin Hong, Sidi Li, Jing Han, Ruibin Luo, Xing Wan, Jian V. Zhang, Ruihuan Xu

**Affiliations:** 1 Clinical Laboratory Department of The Second Affiliated Hospital, School of Medicine, The Chinese University of Hong Kong, Shenzhen & Longgang District People’s Hospital of Shenzhen, Shenzhen, 518172 China; 2 Shenzhen Key Laboratory of Metabolic Health, Center for Energy Metabolism and Reproduction, Shenzhen Institute of Advanced Technology, Chinese Academy of Sciences, Shenzhen, 518055 China; 3 Shenzhen Key Laboratory of Metabolic Health, Shenzhen, 518055 China; 4 Shenzhen Institute of Advanced Technology, Chinese Academy of Sciences, Shenzhen, 518055 China; 5 School of Medicine, The Chinese University of Hong Kong, Shenzhen, Shenzhen, 518172 China; 6 Institute of Pathology and Southwest Cancer Center, Southwest Hospital, Army Medical University (Third Military Medical University), Chongqing, 400038 China; 7 Warshel Institute for Computational Biology, School of Life and Health Sciences, The Chinese University of Hong Kong, Shenzhen, Shenzhen, 518172 China; 8 Department of Clinical Laboratory, Longgang District Central Hospital of Shenzhen, Shenzhen, 518116 Guangdong China

**Keywords:** Computational biology and bioinformatics, Cellular signalling networks, Data mining

## Abstract

The patterns of communication among different chondrocyte subtypes in human cartilage degeneration and regeneration help us understand the microenvironment of osteoarthritis and optimize cell-targeted therapies. Here, a single-cell transcriptome dataset of chondrocytes is used to explore the synergistic and communicative patterns of different chondrocyte subtypes. We collected 1600 chondrocytes from 10 patients with osteoarthritis and analyzed the active communication patterns for the first time based on network analysis and pattern recognition at the single-cell level. Manifold learning and quantitative contrasts were performed to analyze conserved and specific communication pathways. We found that ProCs (Proliferative chondrocytes), ECs (Effector chondrocytes), preHTCs (Prehypertrophic chondrocytes), HTCs (Hypertrophic chondrocytes), and FCs (Fibrocartilage chondrocytes) are more active in incoming and outgoing signaling patterns, which is consistent with studies on their close functional cooperation. Among them, preHTCs play multiple roles in chondrocyte communication, and ProCs and preHTCs have many overlapping pathways. These two subtypes are the most active among all chondrocyte subtypes. Interestingly, ECs and FCs are a pair of “mutually exclusive” subtypes, of which ECs are predominant in incoming patterns and FCs in outgoing patterns. The active signaling pathways of ECs and FCs largely do not overlap. COLLAGEN and LAMININ are the main pivotal pathways, which means they are very important in the repair and expansion of joint homeostasis. Notably, only preHTCs assume multiple roles (including sender, receiver, mediator, and influencer) and are involved in multiple communication pathways. We have examined their communication patterns from the perspective of cellular interactions, revealed the relationships among different chondrocyte subtypes, and, in particular, identified a number of active subtypes and pathways that are important for targeted therapy in the osteoarthritic microenvironment. Our findings provide a new research paradigm and new insights into understanding chondrocyte activity patterns in the osteoarthritic microenvironment.

## Introduction

Osteoarthritis (OA) is a chronic joint disorder that disrupts the metabolism of joint tissues. It is accompanied by inflammation and bone degeneration, and is closely associated with aging^1–4^. Chondrocytes, along with matrix and fibers, make up cartilage and are the only cell type in cartilage^5,6^. In view of the fact that senescence or degeneration of chondrocytes will result in a loss of cartilage integrity, many studies have focused on the apoptosis of OA chondrocytes in an attempt to find out mechanisms affecting the development of OA from the perspectives of chondrocyte differentiation and growth^7–9^. Chondrocytes differentiate and develop in several stages: quiescent, proliferative, prehypertrophic, hypertrophic, and finally calcified into bone^10,11^.

In recent years, with the application of single-cell sequencing technology in osteoarthritis, more discoveries have been made regarding both the differentiation and the subtypes of chondrocytes^12–16^. Different chondrocytes play different roles in cartilage formation. Existing studies on single-cell transcriptomes have identified seven subtypes of chondrocytes: effector chondrocytes (ECs), fibrocartilage chondrocytes (FCs), hypertrophic chondrocytes (HTCs), homeostatic chondrocytes (HomCs), proliferative chondrocytes (ProCs), regulatory chondrocytes (RegCs), and prehypertrophic chondrocytes (preHTCs)^13,15^. Researchers have identified ProC, preHTC, and HTC as more active chondrocyte groups whose functional characteristics have been extensively studied^13^. ProC present in the proliferative zone of the joint growth plate has the function of preventing hypertrophic differentiation at the top layer, while the differentiation at the lower layer near preHTC and HTC is unrestrained^17–19^. Hypertrophic differentiation can also be regulated by preHTC^20^. Chondrocyte mineralization in the surrounding matrix is inseparable from HTC^20–22^. The roles of different subtypes of chondrocytes have been well studied, but the relationships among them are rarely probed. In particular, little is known about how these subpopulations collaborate and communally promote the development of OA in the arthritic microenvironment and what the common patterns of communication among them are. The communication patterns of these chondrocytes are of great importance for understanding OA homeostasis and repair.

To investigate the communication patterns among chondrocytes, we adopted network analysis and pattern recognition at the single-cell level in an attempt to figure out how incoming and outgoing signals coordinate with one another in chondrocytes. We also performed manifold learning and quantitative contrasts to identify conserved and specific pathways in chondrocytes^23^. For the first time, the communication patterns among different subtypes of chondrocytes at the single-cell transcriptome level are compared, which lays the foundation for a further understanding of the osteoarthritic microenvironment.

## Result

### Relatively active chondrocyte subpopulations of ECs, FCs, and HTCs in osteoarthritis

**Figure 1:**
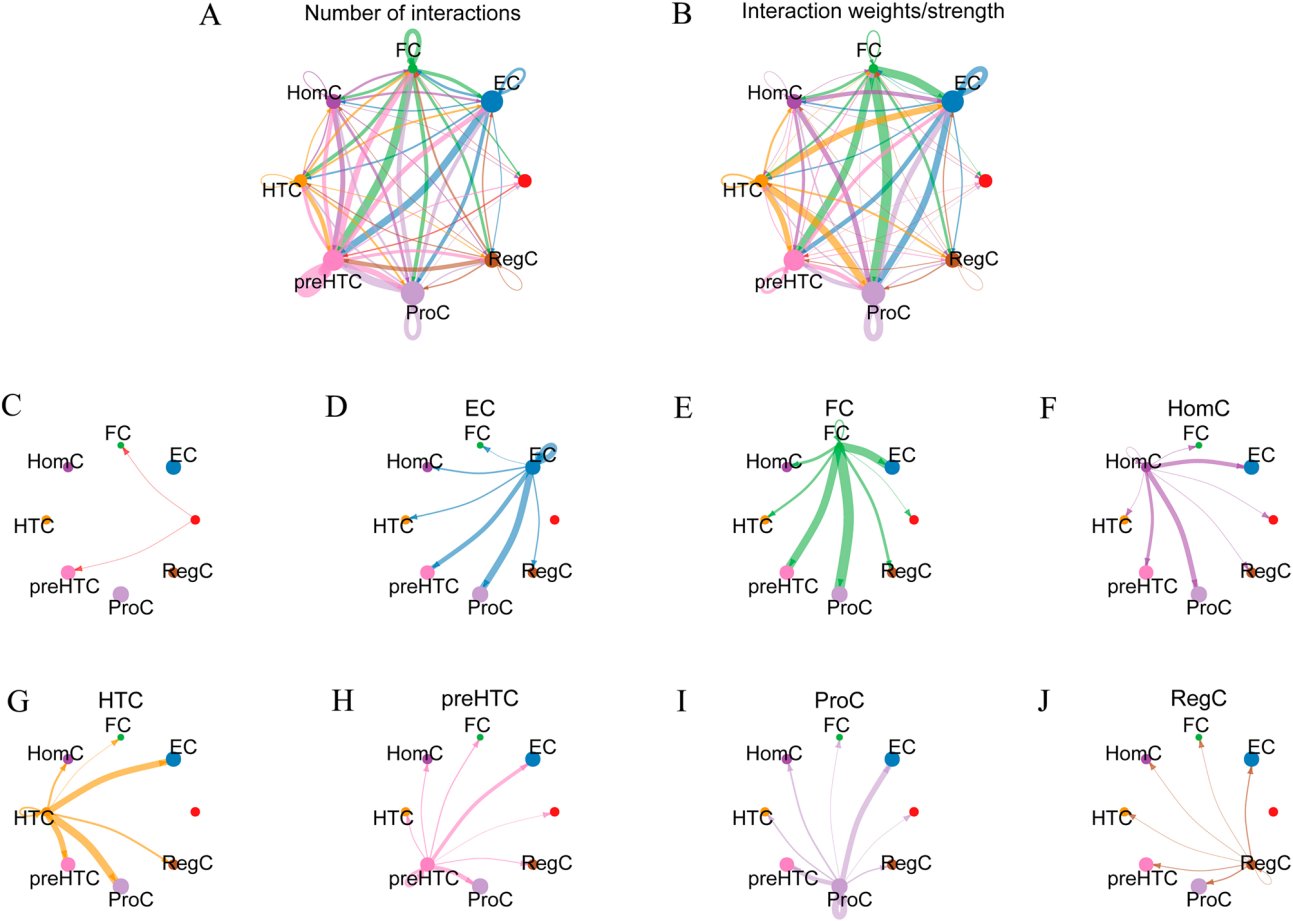
Overview of interactions among chondrocyte subtypes. (**A**,**B**) Total number and intensity of interactions in chondrocytes. (**C**–**J**) Interactions between different subtypes of chondrocytes. (**C**) indicates undefined cell subtypes, (**D**) indicates ECs, (**E**) indicates FCs, (**F**) indicates HomCs, (**G**) indicates HTCs, (**H**) indicates preHTCs, (**I**) indicates ProCs, and (**J**) indicates RegCs.

To investigate the interactions among different subtypes of chondrocytes, we first analyzed the strength and number of interactions among 1,600 chondrocytes. The chondrocytes used in this study were mostly sourced from the knee joints of 10 patients with OA who had undergone knee arthroplasty^13^. Specimens containing all layers of cartilage were individually extracted from the tibial plateau region, following the methodology described in earlier studies^24,25^. The result indicated that the strengths of interactions among ECs, FCs, and HTCs were strong (Fig. 1), whereas those among preHTCs, ProCs, and HomCs were moderate, and those among undefined chondrocyte types and RegCs were weak. Also, the interactions between ProCs and preHTCs were not very strong, but the interactions between HTCs were quite strong. This shows that there are likely transitional relationships between chondrocyte subtypes.

Our results also showed how ProCs, preHTCs, ECs, FCs, and HTCs interact with each other. The close cooperation of these cells is inseparable from their functional role. ProCs are required in the proliferative region of the osteoarticular growth plate; hypertrophic differentiation can be regulated by preHTCs; and the mineralization of the surrounding matrix is inseparable from HTCs^13,26–28^. These results further confirm the close cooperation of different chondrocytes at the single-cell level.

### Communication patterns of osteoarticular chondrocytes

**Figure 2:**
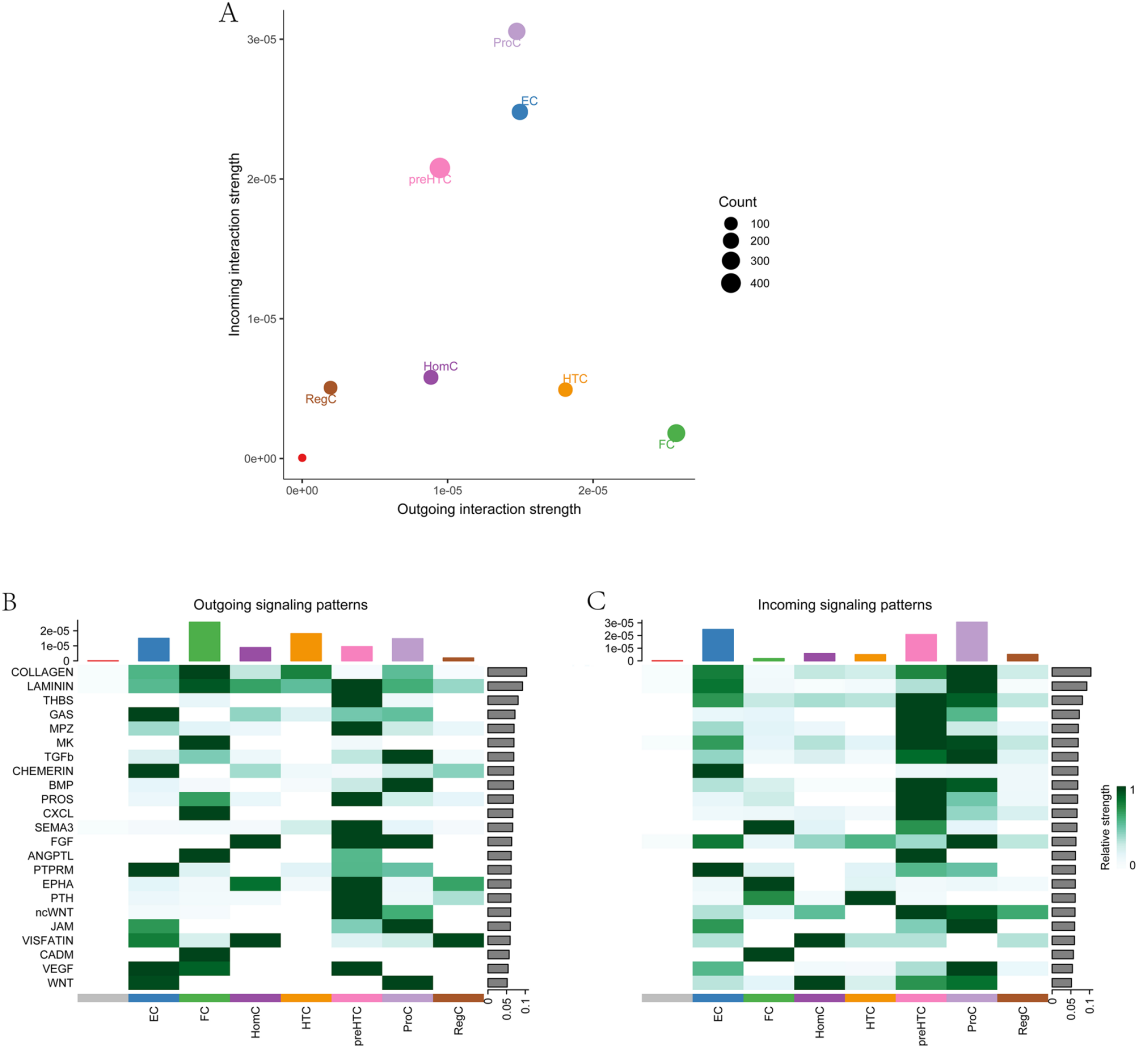
Communication patterns of osteoarticular chondrocytes. (**A**) Chondrocyte subtypes show different strengths of activity in incoming and outgoing signals. The vertical axis indicates the strength of incoming interactions, and the horizontal axis indicates the strength of outgoing interactions. The color of each circle represents a different cell type, and the size of each circle counts the number of cells. (**B**) Outgoing signaling patterns of chondrocyte subtypes. (**C**) Incoming signaling patterns of chondrocyte subtypes. The left side of the vertical axis represents the different signaling pathways, and the right side of the vertical axis represents the relative strength of each signaling pathway. The lower side of the horizontal axis indicates different cell types, and the upper side of the horizontal axis indicates the cumulative signal strength of each cell type.

To explore the communication patterns of chondrocytes, we analyzed the incoming and outgoing patterns of different subpopulations and the signaling pathways occupied by them. By analyzing the communication of different subtypes of cells as a unit, we found that not all cells are active or operate in one mode. Undefined chondrocytes and RegC are “lazier” cell types, while active cell types are: (1) PreHTC, EC, and ProC, which play a predominant role in incoming patterns and a supplementary role in outgoing patterns; (2) HomC, HTC, and FC, which play a supplementary role in incoming patterns and a predominant role in outgoing patterns (Fig. 2A).

The pathways in which different subtypes of chondrocytes are involved vary widely in terms of their degree of activity. Some cell subtypes have overlapping signaling pathways, and some turn off incoming-outgoing signaling pathways, such as undefined chondrocytes (Fig. 2B,C). The undefined chondrocytes have multiple incoming and outgoing pathways that are switched off. HTC is similar to undefined chondrocytes, which have only a few active pathways such as COLLAGEN and LAMININ. This suggests that it does not play an important role in the expansion of chondrocytes and external communication, and that internal regulatory networks may interfere with chondrocyte development (Fig. 2B,C). Interestingly, we found that EC and FC are a pair of “mutually exclusive” cell types, as their active signaling pathways are largely non-overlapping: (1) In outgoing patterns, major EC pathways are GAS, CHEMERIN, PTPRM, VEGF, and WNT, whereas major FC pathways are COLLAGEN, LAMININ, MK, CXCL, ANGPTL, and CADM. (2) In incoming patterns, the main EC pathways are CHEMERIN, PTPRM, and FGF, while the main FC pathways are SEMA3, EPHA, and CADM (Fig. 2B,C). Similarly, we also identified a pair of cell types with many overlapping pathways in ProCs and preHTCs. Basically, all pathways in ProCs overlap with those in preHTCs. These two cell subtypes are also the most active cell types in osteoarthritic chondrocytes. This suggests that the expansion of chondrocytes cannot be achieved without the involvement of these two cell types, which is consistent with previous studies (Fig. 2B,C)^13^.

The above results indicate that the main cell subtypes involved in different signaling pathways are EC, FC, preHTC, and proC. The major pathways of EC and FC do not overlap, and preHTC and proC appear to partially overlap. Regarding pathways involved in cell subtypes, EC and FC can activate different pathways and participate in the proliferative activities of preHTC and proC.

To further illustrate the communication patterns of chondrocytes at different stages, we compared chondrocytes and chondroprogenitors in regard to their communication patterns. The single-cell dataset of chondroprogenitors encompasses various time points during cell differentiation, specifically at days 7, 14, 28, and 42^29^. By consolidating and examining the single cell transcriptome datasets of differentiated cells from these time periods, our objective is to identify overarching patterns in differentiation that can be compared with chondrocytes. We discovered that as the maturation stage progressed, the communication pathways used by chondroprogenitors became stronger, producing more distinct chondrocyte subtypes. (Supplementary Fig. 1A). Some interesting cell subtypes, like HMGB2/CDK1+ proliferating chondrocytes (which belong to ProCs) and CD24/SOX2+ hypertrophic chondrocytes (which belong to preHTCs), had active input signals and moderately intense output signals, even though cells from different datasets all behaved the same, which suggests a high degree of generality (Supplementary Fig. 1A). The number of input and output signals increases along with the number of cell subtypes that develop from chondroprogenitors. Many of these signals overlap with already established chondrocyte signaling pathways, such as MK, ANGPTL, FGF, VISFATIN, BMP, WNT, GAS, TGFb, ncWNT, VEGF, and SEMA3 (Supplementary Fig. 1BC). There are a lot of similar communication pathways between chondrocytes and chondroprogenitors. This suggests that the differentiation of chondroprogenitors and the later function of chondrocytes are molecular processes that cannot be separated.

### preHTC players are in multiple signaling roles

Comparative studies of the chondrocyte pathways were done to learn more about the relationships between the different types of chondrocytes and their pathways, including how they send and receive signals. We found that chondrocytes are involved in 23 signaling pathways, including COLLAGEN, LAMININ, THBS, GAS, MPZ, MK, TGFb, CHEMERIN, BMP, PROS, CXCL, SEMA3, FGF, ANGPTL, PTPRM, EPHA, PTH, ncWNT, JAM VISFATIN, CADM, VEGF, and WNT.

**Figure 3:**
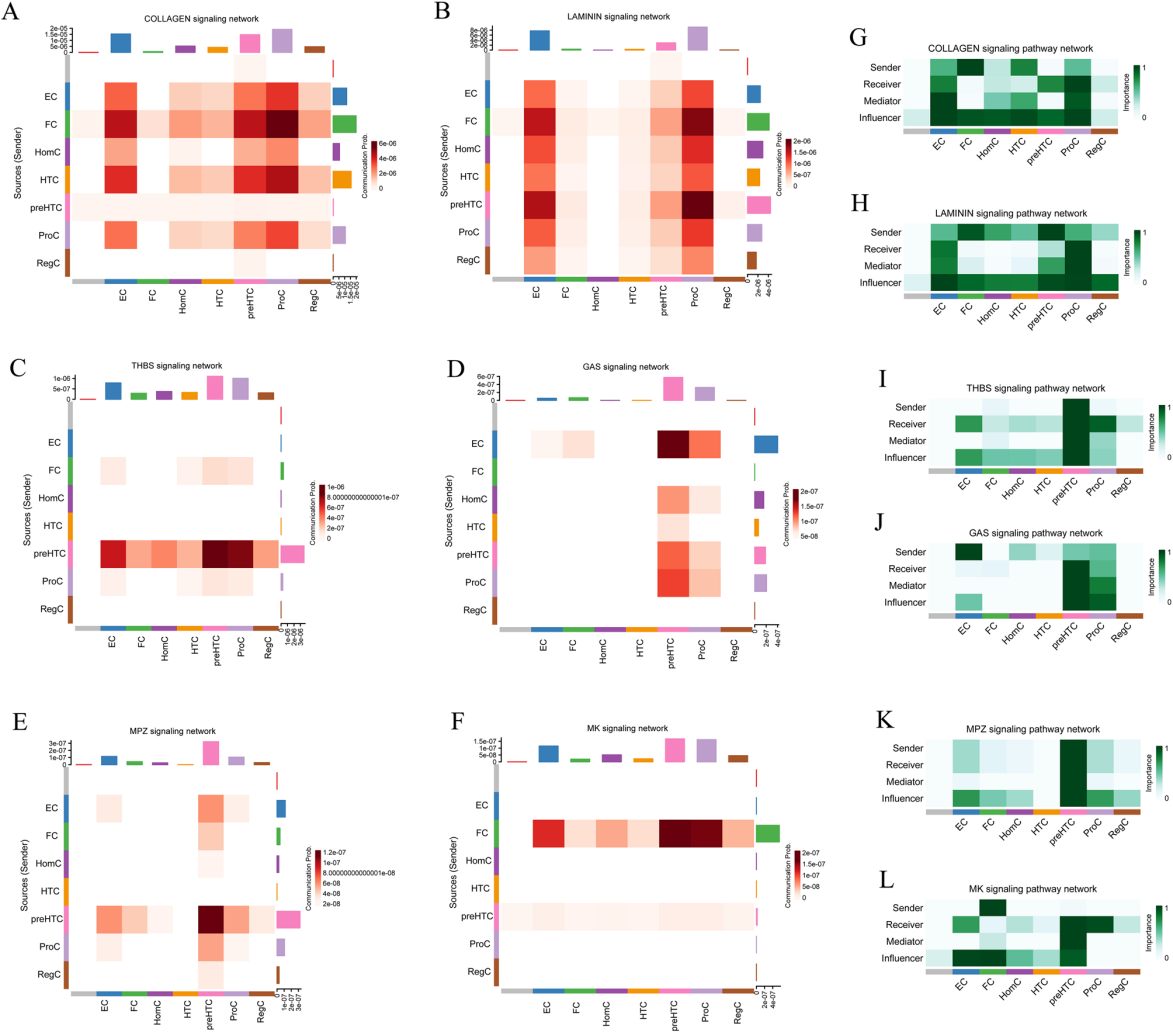
Active pathways in chondrocytes. (**A**–**F**) Interaction strength of chondrocyte subtypes in different active pathways. (**G**–**L**) Communication roles played by chondrocyte subtypes in different active pathways.

We analyzed active pathways (COLLAGEN, LAMININ, THBS, GAS, MPZ, and MK) in cellular subtypes and their roles. Essentially all chondrocyte subtypes act as influencers in COLLAGEN and LAMININ, while EC and proC play multiple roles including sender, receiver, mediator, and influencer (Fig. 3A,B,G,H). ECs and proCs have multiple roles in laminin and collagen pathways, which may be relevant to cartilage development.

We found that proCs were mainly active in COLLAGEN and LAMININ, and that proCs were not more active in other pathways than preHTC. preHTC was involved in different pathways and served multiple roles (Fig. 3A–L). In THBS, GAS, MPZ, and MK pathways, the main active cell subtypes are preHTC and FC, but in terms of the role they play, FC is mainly an influencer and sender in the MK pathway, while preHTC is a sender, receiver, mediator, and influencer in these pathways (Fig. 3C–L).

### Transcript expression levels of ligand-receptor pairs involved in chondrocyte communication and their different cell subtypes

**Figure 4:**
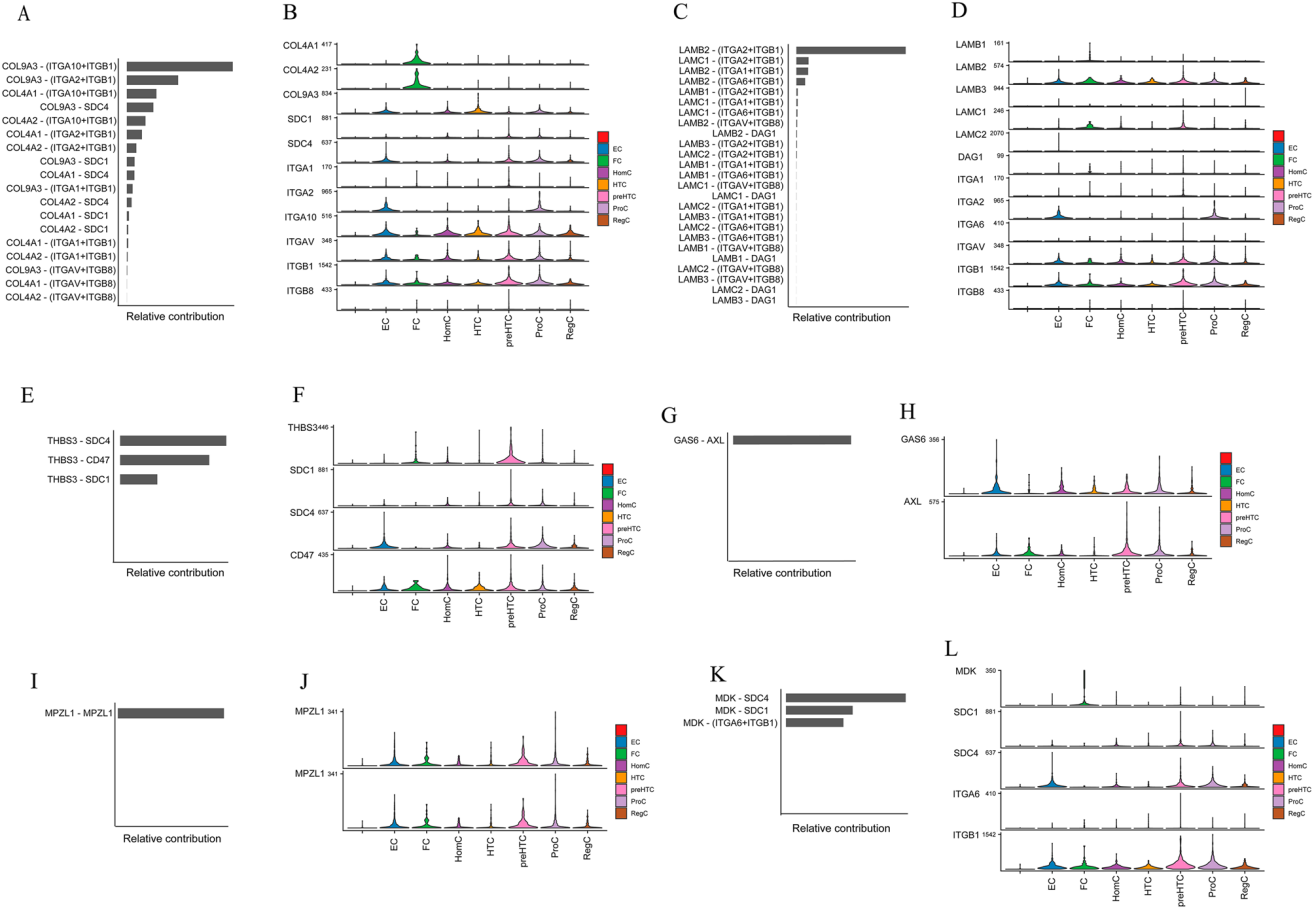
Chondrocyte-active ligand receptors in active pathways. (**A**,**B**) Ligand receptors involved in the COLLAGEN pathway and their single-cell transcript expression levels. (**C**,**D**) Ligand receptors involved in the LAMININ pathway and their single-cell transcript expression levels. (**E**,**F**) Ligand receptors involved in the THBS pathway and their single-cell transcript expression levels. (**G**,**H**) Ligand receptors involved in the GAS pathway and their single-cell transcript expression levels. (**I**,**J**) Ligand receptors involved in the MPZ pathway and their single-cell transcript expression levels. (**K**,**L**) Ligand receptors involved in the MK pathway and their single-cell transcript expression levels.

To explore which ligand-receptor pairs are involved in intercellular communication, we further analyzed their activity in active signaling pathways. We found that COLLAGEN and LAMININ have more ligand-receptor pairs, and that ITGAV, ITGB1, ITGA10, COL4A1, COL4A2, and COL9A3 are the main COLLAGEN pathways (Fig. 4A,B). In the COLLAGEN pathway, active COL4A1 and COL4A2 ligand receptor pairs are mostly found in FC, EC, and HTC cells. Interestingly, ITGAV, ITGB1, and ITGA10 involve multiple chondrocyte subtypes, which suggests that they have a greater impact on the development of osteoarthritis (Fig. 4A,B). In the LAMININ pathway, mainly LAMB2, LAMC1, ITGA2, ITGAV, and ITGB1 are involved (Fig. 4C,D). All of the main types of cells in the LAMININ pathway have active pairs of ligand receptors for LAMB2, ITGAV, and ITGB1. The main cell subtypes in the LAMININ pathway have active pairs of ligand receptors for LAMB2, ITGAV, and ITGB1. LAMC1 is predominant in FC, and ITGA2 is predominant in EC and ProC (Fig. 4C,D).

The remaining active pathways do not have many ligand receptor pairs: GAS and MPZ have only one pair, whereas THBS and MK have three pairs (Fig. 4E–L). Some ligand receptors, such as ITGB1, are widespread in a variety of cell subtypes (Fig. 4A–L).

## Discussion

**Figure 5:**
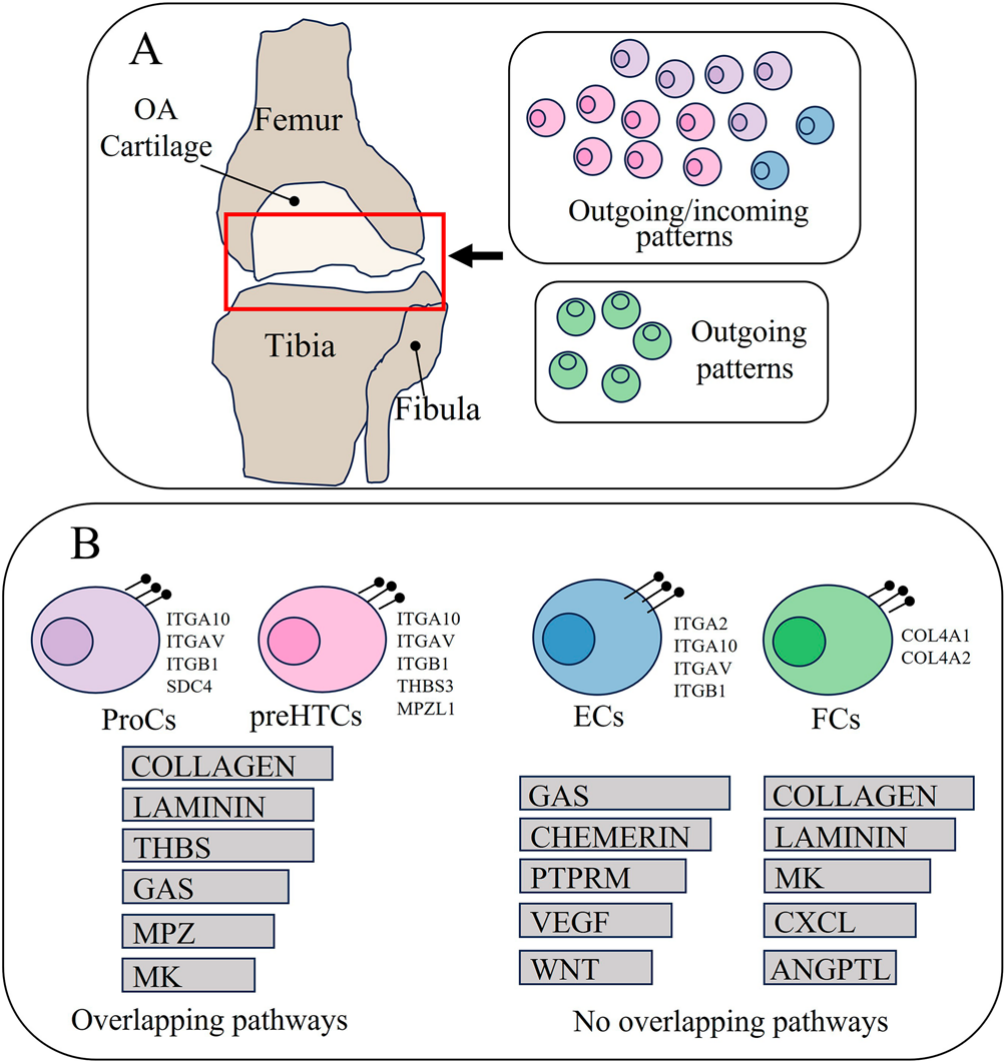
Cellular communication patterns in chondrocytes. (**A**) represents the osteoarthritic knee site and its active chondrocyte subtypes. ProCs, preHTCs, and ECs are mainly involved in the outgoing and incoming patterns, and FCs are mainly involved in the outgoing pattern. (**B**) represents the ligand receptors of the major chondrocyte subtypes and their signaling pathways. ProCs and preHTCs possess multiple overlapping pathways, while ECs and FCs possess distinct pathways.

The communication patterns of different chondrocyte subtypes in osteoarthritis play an important role in the joint microenvironment and provide important references for osteoarthritis development and targeted therapy^9,13^. This study is the first to investigate the activity patterns of different chondrocytes based on network analysis and pattern recognition at the single-cell level (Fig. 5A). Manifold learning and quantitative contrasts were conducted to further identify conserved and specific pathways^23^.

Chondrocyte communication patterns reveal relationships among different chondrocyte subtypes (Fig. 5A,B). Previous studies have not touched upon how the chondrocyte subtypes communicate with one another but often focused on a certain subtype, such as ProC^13^. This study expands the relationships among different chondrocyte subtypes within a communication network. Interestingly, we found that ProC, EC, preHTC, HTC, and FC are more active in incoming and outgoing activities when we cross-talk different subtypes, which is consistent with previous studies in that ProC can promote chondrocyte proliferation and differentiation^13^. ProC is influenced by preHTC and inseparable from EC and FC, which require further mineralization through HTC regulation^17–19^. The relationships among these cells can be probed from incoming-outgoing patterns at the single-cell level, which also provides the basis for targeting different chondrocyte subtypes in the future. We are also the first to explore the role of active pathways and their different subtypes in chondrocytes at the single-cell level. According to our results, preHTCs play multiple roles in chondrocyte communication. ProCs and preHTCs have many overlapping pathways. These two cell subtypes are also the most active in osteoarthritic chondrocytes. Interestingly, we also found that ECs and FCs are a pair of “mutually exclusive” subtypes, of which ECs are predominant in incoming patterns and FCs in outgoing patterns. The signaling pathways they are involved in largely do not overlap. By analyzing ligand receptors and their pathways in chondrocytes, we identified COLLAGEN and LAMININ as the main pivotal pathways. That is to say, they play a very important role in the repair and expansion of joint homeostasis. Osteoarthritic homeostasis requires the promotion and maintenance of multiple chondrocyte subtypes, especially hub subtypes^30^. We found that only preHTC assume multiple roles, including sender, receiver, mediator, and influencer. preHTC ligand receptors are the most active, including ITGAV, ITGB1, and ITGA10.

This study is subject to certain limitations pertaining to the samples used and the presence of various cell subtypes. A dataset consisting of single-cell data from 10 patients was obtained. The distribution of cellular subtypes was found to vary among patients, and there was a lack of grading information for OA, indicating the presence of heterogeneity in the diagnosis of the disease. Further investigation and follow-up studies are required, involving a larger population of patients with osteoarthritis, in order to explore and promote understanding of cellular patterns. Simultaneously, the identification and capture of cell subtypes at the single-cell transcriptional level pose significant challenges in the practical implementation of cell separation techniques such as FACS/sorting. Furthermore, the screening process for subsequent cellular therapeutic applications also requires optimization to ensure accurate capture of the cell subtypes identified at the transcriptional level. During the process of cell expansion, it is observed that various subtypes of cells may demonstrate varying proportions of OA. Therefore, it is necessary to conduct more investigation to understand the dynamics and preservation of chondrocyte proportions at different stages of excavation.

In conclusion, we have looked at their communication patterns from the point of view of cellular interactions, shown the relationships between different chondrocyte subtypes, and in particular found a number of active subtypes and pathways that are important for targeted therapy in the osteoarthritic microenvironment^13,31,32^. The behavior of cell communication is of great significance during clinical application: (1) Cell subtypes with active or more robust communication can be selected as alternative cells for cell therapy. Some of the predominant cell populations we identified in OA, such as ProCs and preHTCs, could be used as alternative cell types for subsequent clinical trials and clinical observations. For example, the identification and input of dominant cells after total joint replacement surgery in OA or the reduction or reversal of the proportion of cellular senescent cell subtypes are beneficial to the repair of arthritis patients. There is also the repair of arthritic stem cells, where the use of our method (using pattern recognition and manifold learning) allows for the rapid targeting of dominant stem cells, thus targeting the selection of cells that have a beneficial effect on arthritis recovery. (2) Interacting relationships between cell ligand receptors, which we can subsequently develop relevant inhibitors to block, interfering with these arthritis-related communications with a view to achieving therapeutic effects. To provide new ideas and therapeutic options for arthritic chondrocytes and their development. (3) In the era of individualized precision medicine, there are a small number of differences in cell subtypes for each individual. By detecting these cell subtype differences, we can use our method to perform rapid screening, reduce errors and discrepancies, effectively deal with the differences at the individual cell level in each patient, reduce experimental financial overheads and time consumption, and improve reproducibility and reliability. (4) It is important to note that the communication patterns among cells in this study are based on single cell transcription in osteoarthritis, with limited information on the spatial distribution of chondrocytes. The future application of single-cell spatial transcriptome and spatial subcellular proteome technologies in osteoarthritis is expected to provide a more realistic picture of the evolutionary trajectory and spatial status of different molecules of chondrocytes during the development of osteoarthritic inflammation^33–39^, and to provide key cell subtypes and their distribution patterns for targeted drug and cellular immunotherapy^32,39,40^.

## Materials and methods

### Projection of cell–cell communication networks

Raw RNA-seq data for OA chondrocyte single cells were obtained from the GEO (Gene Expression Omnibus) database (GSE104782). We used the R package cellchat for cell–cell communication analysis, which required the entering of normalized scRNA-seq mean expression data values and cell annotation information into cellchat to construct a cellchat-compliant object format^23,41^. In order not to affect the number of inferred ligand-receptor pairs, the expression matrix needs to be averaged by default by cellchat's “trimean” gene expression values for each cell group, which is a statistically robust method for averaging gene expression. For signal genes of interest, average expression values were checked using the function computeAveExpr (cellchat, features = c(“COL4A1”, “COL4A2”), type = “truncatedMean”, trim = 0.1). A signal communication network was mainly inferred with the function computeCommunProb, cellchat <- computeCommunProb(cellchat). Individual signaling pathway profiles for ligand-receptor pairs were inferred with the function computeCommunProbPathway, cellchat <- computeCommunProbPathway(cellchat). Single-cell transcriptome dataset of chondroprogenitors and their differentiation from GSE160625.

### Quantification of cell communication patterns

In order to infer the roles played by different cell subpopulations in cell communication (including dominant sender, receiver, mediator, and influencer), we adopted a weighted directed network approach, using cellchat's netAnalysis_computeCentrality and netAnalysis_signalingRole_ network functions for derivation. The parameters are: cellchat <- netAnalysis_computeCentrality (cellchat, slot.name = “netP”) and netAnalysis_signalingRole_network (cellchat, signaling = pathways.show, width = 8, height = 2.5, font.size = 10). Next, different incoming and outgoing signals were calculated by harmonizing cell communication patterns using selectK(cellchat, pattern = “outgoing”) and selectK(cellchat, pattern = “incoming”). Parameters were set to calculate the number of intercellular communication patterns using identifCommunicationPatterns and netAnalysis_river or netAnalysis_dot functions were employed to calculate specific pathways and visualize the assumed signaling patterns. Finally, similar signals were grouped by clustering and grouping to form different sets of signals that facilitate the exploration of heterogeneity between signaling pathways. Functions for signal clustering, grouping, and visualization include computeNetSimilarity, netEmbedding, netClustering, and netVisual_embedding.

### Comparison of cellular communication networks

The number and strength of interactions among single cell transcriptome expression profiles were inferred using cellchat’s function compare interactions. To determine which cell groups showed significant variation in interactions, interactions among different cell groups were compared: par(mfrow = c(1,2), xpd = TRUE) netVisual_diffInteraction (cellchat, weight.scale = T); netVisual_ diffInteraction (cellchat, weight.scale = T, measure = “weight”). The heatmap visualization of the number of interactions and the intensity of the interactions was performed using the netVisual_heatmap function. The netAnalysis_signalingRole_scatter function was used to identify cell populations that differ significantly among data sets in terms of signals sent or received.

### Supplementary Information

Supplementary Information.

## Data Availability

Raw RNA-seq data for OA chondrocyte single cells were obtained from the GEO (Gene Expression Omnibus) database (GSE104782). Single-cell transcriptome dataset of chondroprogenitors and their differentiation from GSE160625.
