# Supplementary material for: Probing the communication patterns of different chondrocyte subtypes in osteoarthritis at the single cell level using pattern recognition and manifold learning
Source: Sci Rep. 2023 Sep 2;13:14467. doi: 10.1038/s41598-023-41874-z (PMC10475121; doi:10.1038/s41598-023-41874-z)
Supplement: Supplementary file 1 — Supplementary Information. [file 41598_2023_41874_MOESM1_ESM.docx]

**
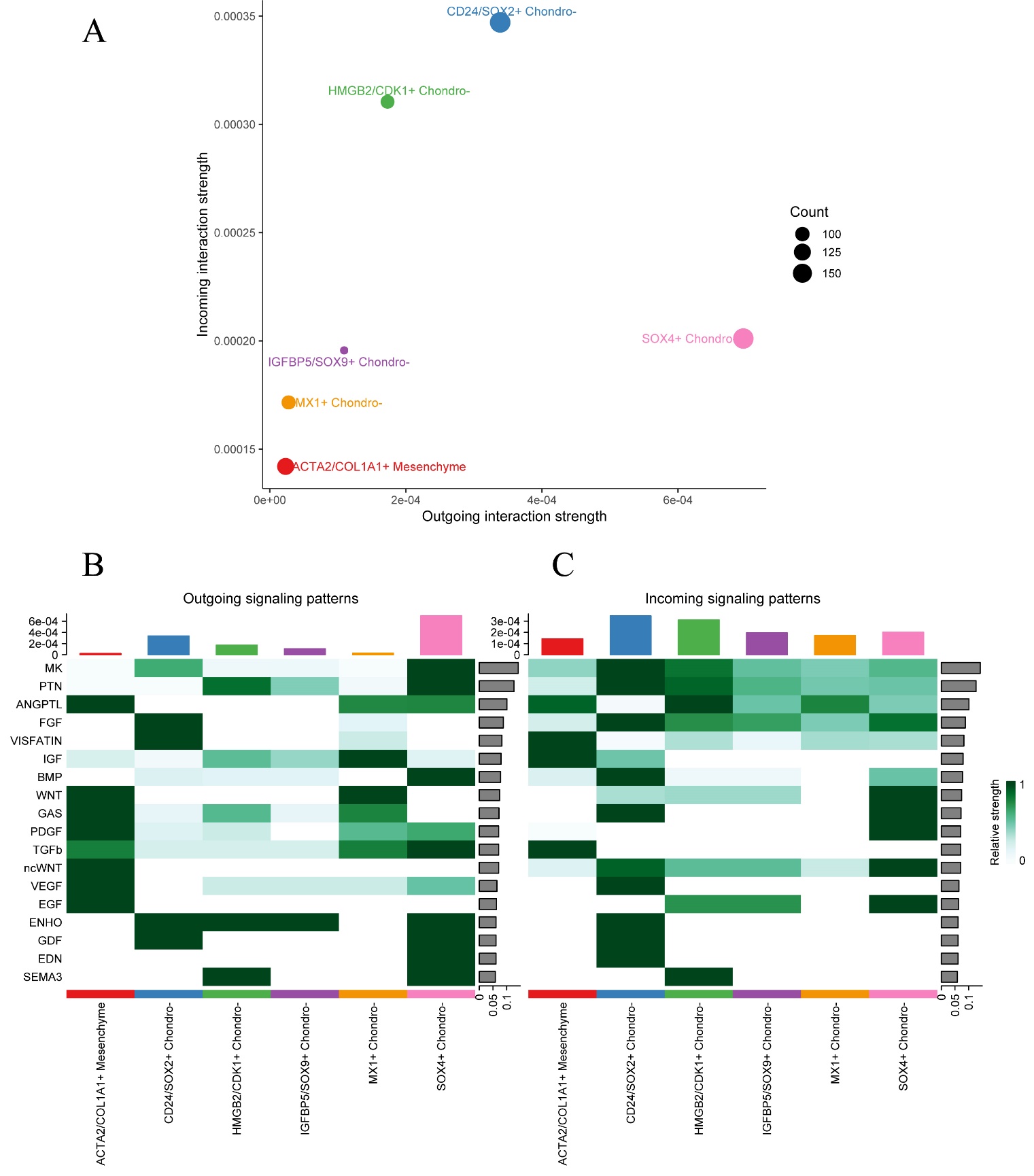
**

**Supplementary Material**

**Supplementary Figure 1 Communication patterns of chondroprogenitors differentiation.**

(A) Chondroprogenitors differentiation shows different strengths of activity in incoming and outgoing signals. (B) Outgoing signaling patterns of chondroprogenitors differentiation. (C) Incoming signaling patterns of chondroprogenitors differentiation.
